# Engagement with behaviour change in people with mild cognitive impairment and mild frailty: a qualitative study

**DOI:** 10.1136/bmjopen-2025-103314

**Published:** 2026-05-04

**Authors:** Tasmin Alanna Rookes, Rachael Frost, Yolanda Barrado-Martín, Jessica Catchpole, Megan Armstrong, Benjamin Gardner, Rebecca L Gould, Claudia Cooper, Cecilia Hammond, Louise Marston, Kate R Walters

**Affiliations:** 1 Department of Primary Care and Population Health, University College London, London, UK; 2 Department of Public and Allied Health, Liverpool John Moores University, Liverpool, UK; 3 School of Health & Psychological Sciences, City St George’s University of London, London, UK; 4 School of Psychology, University of Surrey, Guildford, UK; 5 Department of Mental Health of Older People, University College London, London, UK; 6 Wolfson Institute of Population Health, Queen Mary University of London, London, UK; 7 Public Contributor, University College London, London, UK

**Keywords:** Behavior, QUALITATIVE RESEARCH, Dementia, Cognition, PUBLIC HEALTH, Psychosocial Intervention

## Abstract

**Abstract:**

**Background:**

Many older people experience Mild Cognitive Impairment (MCI), which may compromise the effectiveness of health promotion programmes.

**Objectives:**

We explored engagement with behaviour change among participants scoring 18-25 on the Montreal Cognitive Assessment receiving HomeHealth, a health promotion intervention supporting older adults with mild frailty to maintain independence in England ().

**Methods:**

Of the 46 semistructured process evaluation interviews, 29 participants scored in the MCI range, purposively selected for demographic characteristics and degrees of cognitive impairment and the seven support workers.

**Results:**

Thematic analysis resulted in three themes: Navigating the impact of MCI; Addressing memory as a goal in the intervention; and Adapting Behaviour Change Interventions for MCI. Participants had varied opinions about whether their memory was problematic and whether anything could be done to help. Many reported not discussing memory concerns with support workers. Barriers to engagement in behaviour change included limited social support and not acknowledging memory problems. Facilitators included setting goals which increased or were linked to existing health behaviours, using reminders/prompts and actively involving family members.

**Conclusions:**

Implementing these facilitators into existing and new health promotion interventions delivered to older adults, with suspected but unacknowledged MCI, could overcome the current barriers people with MCI face when trying to engage and benefit from interventions.

**Trial registration number:**

ISRCTN54268283

**STRENGTHS AND LIMITATIONS OF THIS STUDY**

- Interviewing people who were identified for their frailty rather than their memory enabled us to elicit the views of those who, while experiencing some memory impairment, do not discuss their cognitive difficulties with healthcare professionals.
- The participants come from a randomised controlled trial sample and so, the findings may be limited to this specific behaviour change intervention delivered in England and not other health promotion behaviour change interventions, different older populations or other healthcare systems.
- The sample only included adults over the age of 71, and therefore, the findings here may not be representative of younger people with Mild Cognitive Impairment.

## Introduction

 Behaviour change interventions aim to modify behaviours, increase adherence, promote healthy lifestyles and/or improve engagement with clinical services.^1 2^ Mild Cognitive Impairment (MCI) is an intermediate cognitive stage between healthy cognitive ageing and dementia.^3^ MCI is characterised as subjective cognitive complaints and objective cognitive decline, such as forgetting names and past events, which do not interfere with an individual’s ability to carry out daily activities.^4 5^ The prevalence of MCI is difficult to estimate, as it is not routinely tested for in clinical practice and often goes undiagnosed. MCI prevalence in older adults over 65 years’ is estimated to be 15% in the UK.^6^ This prevalence increases as people age, with age-specific reports suggesting an increase from 6.7% in those aged 60–64 to 25.2% in those aged 80–84.^7^ These estimates are in line with other European countries.^8^ While MCI can remain stable or revert to healthy cognitive functioning, there is an increased risk of developing dementia in those with MCI,^5 7 9^ with a reported annual progression rate from MCI to dementia of 5%–10%, compared with just 1%–2% in the general ageing population.^10^

While there are some international guidelines, in the National Health Service (NHS) in the UK there are no specific management recommendations for people with MCI, due to a lack of evidence of effective strategies.^11^ Current guidelines are for healthcare professionals to provide generic health improvement recommendations to all those in later life. These include managing comorbid health conditions associated with an increased risk of dementia and changing behaviours to lead a healthier lifestyle.^12^ However, healthcare professionals report only encouraging physical exercise, cognitive stimulation and socialisation as preventative strategies to reduce cognitive decline and these are rarely followed up on.^13^ Healthcare professionals acknowledge that this approach, with no reinforcement, is unlikely to lead to behaviour change.^14^ Identifying and intervening with health promotion interventions, which help target modifiable risk factors, when an individual has MCI could prevent further decline to dementia and is a potential target for intervention in later life.^9 15 16^

Many health promotion interventions have been delivered to people with MCI to improve their cognitive and physical functioning outcomes.^17^ However, when people with MCI engage with generic health promotion interventions, not specifically designed for people with MCI, some evidence suggests their outcomes are less beneficial when compared with those with healthy cognition for their age.^18^,^20^ Benefits from falls prevention services in cognitively healthy adults, such as reducing the risk and rate of falls, have not been equally translated to those with MCI.^19^ However, this evidence is limited as researchers often do not test for differences in effectiveness from health promotion interventions between those with healthy cognition and those with MCI. This has resulted in a lack of guidelines and recommendations for how stakeholders should adapt and deliver health promotion interventions to older adults with MCI.^18^ We therefore need to understand more about how people with MCI engage with behaviour change and how we can overcome barriers in this population.

This research highlights findings from interviews conducted with participants with MCI who received a behaviour change intervention to support older adults with mild frailty to maintain their independence (HomeHealth—ISRCTN54268283—Post-results), as part of a randomised controlled trial (RCT).^21^

The HomeHealth intervention, developed based on the COM-B model (which suggests people need the Capability, Opportunity, and Motivation, to change Behaviour),^1^ included many elements of behaviour change for health promotion in older adults, utilising an asset-based approach.^22^ Participants were offered up to six one-to-one sessions delivered over 6 months with a trained support worker assisting participants to identify and work on person-centred goals, around mobility, nutrition, psychological well-being and/or social inclusion, setting action plans, reviewing goal progress and problem solving barriers. Goal progress was reviewed at subsequent sessions to aid behaviour change, through problem solving, maximising motivation, action planning, coping with setbacks, habit formation and maintenance.^21 23^ MCI was not the focus of this intervention, but individuals could set goals around this if they wished. Over 60% of the sample scored within the MCI range using the Montreal Cognitive Assessment (MoCA).^24^ This work was an additional study linked to the process evaluation of the HomeHealth RCT, which was conducted to understand the mechanisms of impact and implementation of the intervention.^25^ The analysis in this paper explores participants with MCI and the support workers delivering the intervention’s experiences of how memory issues impacted engagement with and perceived benefit of receiving the intervention.

## Aim

To explore how people with MCI engaged with and perceived themselves to benefit from a behaviour change intervention (HomeHealth) for people with mild frailty, from the perspectives of both those delivering and receiving the intervention.

## Methods

This study has been reported in line with the Standards for Reporting Qualitative Research,^26^ see online supplemental table 1.

### Eligibility

Participants were recruited into the trial from three regions of England, London, Hertfordshire and West Yorkshire. Participants interviewed for this study were approached if they had taken part in the intervention arm of the HomeHealth trial receiving the health promotion service^21 23^ (community dwelling, aged 65+, mildly frail^27^ and scored as having potential MCI (MoCA score 18–25 at baseline and/or 12-month follow-up). Participants were purposively sampled for a range of diverse MoCA scores and participant characteristics, including age, gender, ethnicity, geographic location, socioeconomic status (linked indices of multiple deprivation from participant postcode), education level and differing type of goal(s) participants chose to set across the four main goal domains. In the intervention group, 125 out of 195 participants were categorised into the MCI group, 53 of which were invited to interview, of whom 29 accepted and were interviewed (54.7%). In addition, seven health promotion (HomeHealth) support workers who were trained and employed in the voluntary sector to deliver the intervention were invited to interview, all of whom consented to participate.

### Data collection

One-to-one semistructured interviews were conducted with 28 participants and one participant-carer dyad and seven support workers. All interviews with people with MCI were conducted in their homes except for two, which were conducted remotely via videoconferencing. Interviews were conducted by four researchers (TAR, JC, YB-M and one additional team member). Interviews lasted for an average of 62 min (range 35–98 min) and were carried out between August 2022 and May 2023. All interviewers had completed qualitative interview methods training, had no prior connection with the participants and kept reflective diaries after each interview and throughout the interviewing process.

The topic guide was developed with the HomeHealth RCT process evaluation team.^25^ Alongside the process evaluation topics, covering experiences of the intervention, goal setting, support worker and generic barriers, those with MCI were asked additional questions about their experience of memory difficulties. Interviews opened asking participants about how cognitive impairment was impacting them, to help with probing later in the interview. The interview then covered the goals participants set, their progress towards these goals during and after the intervention, and how cognitive impairment may have impacted these. To support people with memory difficulties to participate in the interview, a summary of the goals participants set and a picture of the support worker who visited them was provided to prompt their memory.

The interviews with the intervention support workers, conducted as part of the process evaluation, also included questions for this study to explore their perception of how MCI impacted participants’ engagement with and perceived benefit from the intervention.

Data were analysed alongside participant interviews to provide a deeper insight into barriers, facilitators and ways to overcome these. Interviews were audio recorded and transcribed by an external company. Data accuracy was checked, and transcripts anonymised by the research team.

### Patient and public involvement

Two public contributors with MCI were involved in the project throughout, meeting five times over 1 year, assisting with finalising the research questions, developing and updating the topic guide, and interpretation of themes.

### Data analysis

The initial approach to analysing the whole dataset used codebook thematic analysis to facilitate a team-based approach with several researchers coding the interviews and different parts of the data.^28^ The foundation of the codebook was created by two researchers (TAR and YB-M) independently coding 10% of transcripts, followed by discussion and refinement with the rest of the team. This initial coding, conducted in NVivo V.12, resulted in inductive themes derived from the whole dataset which were then iteratively grouped and synthesised through team discussions (TAR, JC, YB-M and RF). For more information, please see the process evaluation papers.^25 29^

Transcripts from interviews with people with MCI only were analysed using reflexive thematic analysis, following an inductive approach.^28 30^ TAR immersed themselves in the dataset and generated initial codes capturing participants’ discussions and experiences related to cognition. Coding and theme development progressed iteratively, with codes being refined, expanded or merged as analytic insights deepened. Developing patterns were summarised into initial themes and reviewed against the dataset to ensure coherence and clarity. Theme construction was supported through ongoing discussion and reflexive dialogue within the research team. The research team brought perspectives from clinical trials, public health and psychology, with shared interests in ageing and well-being. These disciplinary backgrounds inevitably shaped interpretive decisions, and the team engaged in ongoing reflexive consideration of how their assumptions and professional positions might influence the analysis.

## Results

### Participants

Participants with MCI had a mean age of 81.7, more than half of the participants were female. Baseline MoCA scores had a mean of 21.7, which slightly increased at 12-month follow-up to a mean of 22.6 (see table 1). All support workers were female, but no additional characteristics were collected.

**Table 1:** Characteristics of participants

Characteristic	Interviewed (n=29)	Mean (SD)
Age range	71–94	81.7 (6.4)
Gender (% female)	17 (59%)	N/A
Ethnicity (% White British)	18 (62%)	N/A
Socioeconomic status: Index of Multiple Deprivation (IMD) range*	1–10	4.9 (3.0)
Baseline MoCA score range	16–26	21.7 (2.8)
12-month MoCA score range	17–28†	22.6 (3.1)
Support from unpaid carer (%)	23 (79%)	N/A
Goals set	37	N/A
Mobility	19	N/A
Psychological	5	N/A
Social	3	N/A
Nutrition	1	N/A
No goal set	5	N/A

* IMD calculated by ranking geographical areas by relative deprivation, using factors such as income, employment, health, education, housing and crime^43^

† n=26: MoCA not completed at 12-month follow-up for three participants interviewed.

MoCA, Montreal Cognitive Assessment; N/A, not applicable.

### Thematic overview

To encompass the experiences of people with MCI engaging with behaviour change and whether they perceived they had benefited from the intervention, we constructed three overarching themes: (1) navigating the impact of MCI; (2) addressing memory as a goal in the intervention and (3) adapting behaviour change interventions for MCI.

#### Theme 1: Navigating the impact of MCI

##### Impact of cognitive impairment on daily activities

Participants reported cognitive impairment issues centred around getting out and about, including driving, physical and mental slowness, going on holiday, doing their hobbies and attending support groups. Impacts of cognitive impairment on executive function included difficulties with concentration and slower memory retrieval.

I was standing talking about something and I stopped dead in a conversation, I had forgotten what I was talking about, and nine times out of ten it doesn’t come back to me until later. 02138*,* Female, 70s

Cognitive impairment also impacted people’s working memory, impairing their ability to learn new things and process short-term memories into long-term memories. These included remembering new people’s names, events, appointments, or to take medication, and learning how to use new or changing technology.

It actually feels strange that how come that every day I can remember and then all of a sudden on the day it needs to be [remembered], I totally forgot. You know, it was only because I looked in my diary and I forgot where my diary was but then I remembered I put it in my handbag. 02138, Female, 70s

When participants experienced these difficulties, they often reported negative feelings such as annoyance or embarrassment that affected their mental well-being and confidence to engage in social interactions.

Yes, very embarrassing when you meet someone and you have to say, “Oh hello, how are you?” Rather than “Hello so and so.” It’s really embarrassing. 02021*,* Male, 80s

I’ve always been a very confident person but I’m not so confident anymore and that makes me angry. I’ve always had a good memory. 0305*6,* Female, 70s

##### Existing strategies for managing cognitive impairment

Many participants had existing strategies in place to support their memory, which were encouraged by the intervention support worker.

You’d go through the tips, tricks, suggestions around equipment and aids, and they’d discuss with their GP. They’d have blister packs, for example. They’d have reminders on their phone. They’d buy wall calendars. And kind of set goals around having one location where they keep things, and trying to build habits around that, and making sure they have notebooks by the phone. And kind of following up to see how they’re getting on with that. Support worker 2

These included calendars and diaries for upcoming appointments, writing notes of important things to remember, using alarms and technology to prompt or remind about completing a behaviour.

[My family] persuaded me to get a smartphone to have a diary on your phone, and also to have a timer which is a reminder to do whatever it is… I’ve got something I have to do this afternoon at half past five, so I’ll set my timer quarter past five and when it goes off, I’ll have a look in my diary and see what the hell that was about. 01023*,* Male, 90s

Other existing strategies included doing cognitive exercises to keep the mind active, which they felt was important to remember to do daily activities.

Well, I was thinking that my wife, she died of Alzheimer’s. And I don’t think I’m going that way. I do the puzzles, the crosswords and the codeword. I don’t think there’s much change in that, I usually get them out eventually. But the doctor said if you can do the puzzles then you haven’t got Alzheimer’s. 01088, Male, 80s

### Theme 2: Addressing memory as a goal in the intervention

#### Conceptualisation of memory to motivate change

Whether and how participants conceptualised their perceived memory abilities varied and influenced their motivation to address memory as a possible goal. Some felt they had no problems with their memory at all.

I remember what I have to remember. It’s only now and again I forget things. 02111, Male, 80s

However, most participants struggled with cognitive impairment impacting various aspects of their life. Of these, some acknowledged declines in their cognition but attributed it to normal ageing and not therefore something that could (or should) be addressed.

I suppose I’ve had [a memory] problem for maybe a couple of years [whereby] you just forget what it was you were going to do. But from what I've read here, there, and everywhere that’s not dementia, it’s just old age. 02107*,* Female, 80s

Others were aware that their cognition was worse than it should be but felt nothing could be done to improve or manage their cognitive impairment.

Yes, I do definitely have memory problems, I can’t remember names, I have to keep thinking. Or sometimes can’t remember the shop names. Things are becoming worse and worse. And remembering things is becoming most acute, really badly. There isn’t much I can do… I don’t know how else I can remember. 02021, Male, 80s

Finally, a smaller group of participants acknowledged their cognitive impairment and wanted to work on it.

So, in the long-term I just need to make sure that I've got the mobility and don’t get into a stage of dementia… because I don’t want to lose my independence. It’s very important, if I lose my independence I'll just finish. 02107, Male, 70s

Participants varied in their willingness to discuss their memory with the support worker. Some participants were reluctant to discuss their memory, as they felt they had no problem with their memory, it was not impacting them in a meaningful way or that the memory problems they were having were part of normal ageing.

Nothing that impacts on a daily basis, just silly things like going into a room, very, very occasionally, and you just think, ‘oh what am I doing here’, then I remember, and you do it. So, nothing that I think is out of the ordinary. 01122*,* Female, 70s

Others were aware that their memory was worse than it should be but felt like nothing could be done to improve it, as they felt the difficulties were inevitable with old age or felt that the support worker could not offer anything that would help.

I’ve got all kinds of other problems, but [the support worker] was unable to give me my hearing back or restore my short-term memory or any of the other problems of old age, which are what happens. 01023, Male, 90s

#### Requirements for setting memory-based goals

If support workers did not explicitly discuss memory as a goal, then participants were unlikely to start the conversation around this, unless they felt it was impacting their daily life or that there was something that could be done to help.

Had she anything to offer [for my memory], she would have told me, and she didn’t. 01023*,* Male, 90s

Some reflected in the interview that they would have liked to discuss their memory but felt contacting their general practitioner (GP) was the only avenue to do this. It seemed some participants lacked confidence in the support workers’ ability to support cognition, even though support workers received training around this, and participants could set a memory goal.

I don’t think she was really able to [address memory]… If you understand me. I don’t think she was clued up on that part of it. And that’s no offence to [the HomeHealth worker]. 03108*,* Male, 70s

This was reflected in some of the interviews with the HomeHealth support workers, who felt that additional training to help people with MCI to both set and make progress towards their goals would have been valuable, especially as their cognition became more severe and progressed towards dementia.

I think more training around the approach for goal setting for people with cognitive impairments, or when dementia started to progress more, would be valuable. Support worker 1

This highlights a disconnect between what is available and where participants think they can access support in relation to their cognition. As the intervention focused on a broader approach and maintaining functioning, some participants stated that they did not know what to expect from the intervention nor what success would look like, despite this being explained to them at the outset.

She wasn’t able to tell us what help we would expect from her. We weren’t sure why she was coming. 03056, Female, 70s

### Theme 3: Adapting behaviour change interventions for MCI

Almost all participants set at least one goal in the intervention period, and choice of goals and motivation to achieve them did not appear to be influenced by level of cognitive impairment. Those who had progressed to dementia since baseline had issues setting and working towards their goals, where support workers took a more case management approach. This was particularly important when the participant did not have a carer or family member available to advocate for them.

One gentleman that I worked with I noticed that he started getting really, really forgetful and that was one of the concerns I had with him and I rang the GP up and when I said the Community Matron referral happened, I did a joint visit with the Community Matron to this gentleman and he, she did a small, like a short memory test with him and obviously there was concerns and you know and she thanked us for bringing him to their attention because he does need that extra help and support. Support worker 2

#### Techniques to adapt behaviour change to memory impairment

Some participants who acknowledged their cognitive impairment highlighted that it was difficult for them to identify the goals they needed to work on. For these participants they needed more guidance and direction from the support workers as to what goals they could work on.

[Memory difficulties make it harder] to decide what to do. You sit with an empty page. You want to make a plan how you’re going to approach it but then the mind doesn’t work. 02037, Male, 80s

Participants were more successful at achieving their goal or making progress when they had a clear action plan of how to adapt their existing behaviour to align with their goals.

We would sit down and have a plan and said what we we're going to do today. Two or three things. And then we’ll see how much progress I can make. And there is a few things she suggested, particularly for the memory thing, where the activities are for various things and in regard to everything. 01114, Male, 70s

Also, pairing a new behaviour with something they were already doing also increased participants’ ability to do the new behaviour. This method increased participants’ ability to embed the new or adapted behaviour into their daily routine. This enabled them to maintain their behaviour change when they might have forgotten to do it, when motivation is low or if physical health and mobility barriers arose.

But I went to physio, and I still do my exercises. I did them this morning when I got up. I have one of those squeeze balls which I use in the morning, two or three times a day… So, it’s really a case of getting up in the morning and getting the stretching exercises going. I do these things while I’m watching telly. 03038, Male, 80s

Also, support workers built on existing strategies participants used for managing their memory to enable them to engage with the intervention, such as using calendars, alarms, reminders and liaising with family members and friends.

Until you've met someone, you don't know that they've got to write [the appointment] on the calendar. Once it was on the calendar, he was always there… So, getting to see him was quite a challenge at the beginning, because even if he got a letter, [he] hadn’t written it on the calendar. I turned up he wasn't there. Once I’d met him once and I [ensured] the calendar was written on, he was there every single time so yes, again he already had his technique. Support worker 1

When written down action plans were not in place for people with MCI or participants did not follow them, they struggled to bounce back from setbacks.

No, I like to carry on whatever I can rather than chalking new goals, in my situation it’s difficult to plan anything from day to day. 02021, Male, 80s

Well, I was doing some exercise classes, and I couldn’t cope with them while I was ill, and it has been very difficult. I cannot do it now. I’ve been trying to do just five minutes of things like that, but I become very, very tired while I do the exercises. 01102, Female, 80s

For the participants with MCI who had faced a setback, skills around coping with setbacks, pacing based on current energy and cognitive functioning levels and suggesting an alternative goal that may have higher motivation helped them to engage more and increase the perceived benefit.

Now I rotate between walking, swimming. I’ve not really been in the gym, because of the back issues. But today I was swimming, and I was talking to my coach, and he was saying that probably it would help if I started going on the treadmill. I think the whole thing is just to keep on moving. That’s the message, you know. And so, I’ll see if I can do 10–15 min on the treadmill to start with. 01114, Male, 70s

#### External support needed to engage in behaviour change

Participants with MCI relied on the support worker as an external motivator to remain engaged with the service and the behaviour they were trying to change. They acted as a reminder and someone external who was monitoring their progress.

It’s just always good, you know, when somebody’s there, just to remind you. And if you made a commitment, then just to check to see whether you’ve done it or not. 01114, Male, 70s

Some participants wanted contact in between the monthly sessions to act as a reminder or prompt as to what they should be doing and to help problem solve when barriers arose between sessions. They also wanted booster sessions beyond the 6-month intervention period, for the support worker to check on their goal progress and to provide support with new problems.

Yes, I think just checking on me and all the progress I’ve been making would be helpful. 01051, Male, 70s

Without this additional support being provided, some participants highlighted that they could not remember what they had planned to do and when, which impacted the amount they could engage with and maintain the benefits from the intervention.

Sometimes I will forget to do a particular exercise. For instance, bending down trying to touch my toes is not very easy. But when I was doing the exercises, I could almost reach my toes. But if I forget for a week or so, then it feels difficult. Then I say, ‘oh I didn’t do it regularly’. 02021*,* Male, 80s

Many participants relied on support from family and friends to be able to engage with behaviour change and maintain these changes over time, such as having someone to remind them to complete the health-promoting behaviour and support them using calendars and prompts. This support came in different forms. For some participants, having someone to go out into the local community with encouraged them to get out to do the things they needed to do or socialise with others.

At the blind club that I go to, there’s a volunteer there, and… she does come out with me, and she takes me on buses and gets me used to routes, which is good, so that I know when to get off a bus, and she’ll take me to the supermarket. So, I do have that help really, so that’s good. 01122*,* Female, 70s

Other participants needed more practical support to help them to remember to complete the behaviour, such as eating well.

Yes. I do discuss it with my wife… she’s careful about what I eat. In case I forget. She reminds me. 01051*,* Male, 70s

However, relying on others was a barrier when this support was not available and some participants felt worried for the future if something were to happen to their support network.

They opened somewhere else where [my friend] can’t take us anymore because Thursday is her busy day… [You] have to rely on other people it’s not just yourself. 02138*,* Female, 70s

## Discussion

Through qualitative interviews, we highlighted the perceived barriers and facilitators for people with MCI engaging with and benefitting from a health promotion behaviour change intervention for older adults with mild frailty. Barriers to engagement for people with memory difficulties were limited discussions around memory in the intervention, not acknowledging memory as a problem and a lack of social support.

In some cases, these barriers were reportedly overcome by identifying existing strategies to manage memory difficulties and embedding these into action plans and pairing new behaviours with existing behaviours to embed into their routine. Facilitators also included engaging existing support networks in behaviour change where possible and if not, providing active support to navigate healthcare systems and access local support services. Some lacked motivation as they felt nothing could be done and education that cognition is to some extent modifiable and may be addressed as a relevant part of a broader health promotion intervention for older adults may increase engagement. Support for the maintenance phase of behaviour change, with ongoing less frequent support to increase their internal motivation and provide support when new barriers arose might enable sustained behaviour change.

### Results in context

In line with recommended person-centred care for people with dementia,^31 32^ the unique barriers to engagement for each person with MCI receiving an intervention should be identified, which might include lack of acknowledgement of MCI or its impact, social support and self-confidence. This aligns with the COM-B model of behaviour change, which this intervention was based on, where psychological capability and social opportunity are necessary to enable people to change their behaviour.^1^ Our findings suggest that even when this need is highlighted in training of support workers, MCI might not be openly acknowledged in services where this is not the focus, prohibiting explicitly addressing these barriers.

To help support workers to identify and overcome these barriers, specific training around MCI and the associated needs should be considered, as some support workers would have valued more support and training to address memory concerns. In a recent umbrella review, the need for ‘well-trained, knowledgeable and experienced healthcare professionals/support workers’ was emphasised to facilitate participation in physical activity interventions in people with MCI and dementia.^33^ The approach support workers take to support people with MCI is important, due to some people not acknowledging their declining cognition. For those who deny the presence of cognitive impairment or minimise its importance, support workers may need to adapt their approach to tackle MCI as part of a health promotion intervention without explicit discussions, such as suggesting small practical strategies to overcome barriers related to unacknowledged MCI e.g., writing appointments on a calendar or setting a reminder to do the chosen health promotion behaviour, linked to a well-established activity.^34^ These strategies should build on existing ones the person is already doing, to encourage and increase goal setting and progress. For those who do not want to talk about or set a memory-focused goal, support workers need to be able to support individuals to embed strategies that overcome any barriers due to cognition within the goals and action plans which participants choose to set, rather than as its own independent goal.

Person-centred goal setting in older adults to reduce dementia risk has shown improvements in physical and cognitive activity and other health domains,^35 36^ however in our study many participants with MCI were not aware of these modifiable risk factors. It is important that people engaging with health promotion interventions understand that memory and cognition is something they can address. This includes both incorporating adaptations into their daily routine to manage current memory difficulties and incorporating new healthy behaviours, accounting for memory. Currently, public knowledge of modifiable risk factors for cognitive impairment, such as physical inactivity, poor nutrition and medical conditions, is low.^37^ Public health campaigns to inform people of the modifiable risk factors to prevent cognitive decline and improve health more widely are likely to increase proactive engagement in health promotion behaviours and motivation to change behaviour to reduce the risk of developing dementia.^38^ This is supported by 62% of people self-reporting initiating at least one health promoting activity following the diagnosis of MCI and discussion with a clinician.^39^

When delivering health promotion interventions to people with cognitive impairment, it is beneficial to target behaviours which build on or can be linked to existing behaviours that are already part of people’s daily routines (e.g., performing exercises while waiting for the kettle to boil), rather than introducing something new. This is due to declining executive skills, which make setting goals and following a plan difficult.^40^ When comparing our findings from those with MCI to those with healthy cognition interviewed for the wider process evaluation of the HomeHealth intervention, we see that this pairing of behaviours and action plans to overcome barriers seemed a particularly important facilitator to engagement with behaviour change in those with MCI.^25 29^

Those with MCI may be less likely to benefit from behaviour change interventions in comparison to those with healthy cognition, which could result in them engaging less. One systematic review in people with type 2 diabetes found worse cognition was associated with more difficulties in self-management.^41^ Our findings suggest people with MCI can still undertake health promoting activities, but external support played a much larger role. External support therefore needs to be built into future behaviour change interventions when including people with MCI. It may also be valuable to explain and remind participants of the purpose of the intervention on a regular basis, and if external support is not available then facilitate engagement with other appropriate services.

### Strengths and limitations

The study was conducted within a national RCT in three diverse sites with an experienced research team. Interviews were conducted with participants with mild frailty who had received an intervention focused on behaviour change aiming to maintain independence. Therefore, the findings are limited to the insights from one specific behaviour change intervention and may not be applicable to other health promotion behaviour change interventions or different older populations, for example those without frailty. However, barriers and facilitators to overcome these are in line with findings from other physical activity interventions delivered to people with MCI.^33^

This qualitative study relied on the sample of an RCT, who were generally White British and educated to a good level. This may limit the transferability of the findings to people who are less likely to participate in an RCT and to those from other ethnicities. Also, while MCI prevalence increases with old age, onset can occur a lot earlier. Participants had to be over 65 to take part in the RCT, and nobody under the age of 71 was interviewed as part of this study. Therefore, the findings here may not be representative of younger people with MCI or those in the earlier stages of cognitive impairment.

To identify those who have MCI but are not diagnosed, MCI was rated using participants’ MoCA scores, as a proxy measure of MCI, which has both strengths and limitations. On the one hand, the MoCA is not a diagnostic tool and without clinical judgement it cannot confirm whether a participant would be classed clinically as having MCI. Some included participants may not have MCI, and instead their low MoCA score due to other reasons, such as lack of sleep, low mood, or a major life event. While the pragmatic decision to use the range 18–25 is supported by some literature, there is other literature which suggests a more conservative range 21–26. However, the study aimed to understand the experiences of people who do not have a diagnosis of MCI and are unlikely to seek help until their cognitive difficulties have progressed and wanted to include as wide a range of perspectives as possible. Screening tools, like the MoCA, provide a unique opportunity to understand the cognitive difficulties the general mildly frail population face, outside of the clinical setting. Therefore, a wider range of people’s views and experiences were included, rather than just of those who seek help and have a diagnosis. In addition, the MoCA was developed with the purpose of identifying and screening people with early signs of cognitive impairment and has good construct validity for assessing MCI.^24 42^ In addition, a couple of participants scoring within the MCI range at baseline had received a dementia diagnosis by the time of the interview, and their experiences may differ from those with MCI.

### Implications for research and practice

Given that up to 20% of people aged 65 years and over have MCI, behaviour change interventions promoting healthy ageing should account for these differences in cognitive abilities. Those who develop new and/or deliver existing healthy ageing interventions should adapt their approach and design to account for MCI. This may include training service deliverers to be aware of the signs of cognitive impairment, ways to adapt their goal setting approach, providing additional prompts and reminders, engagement of external support in new behaviours, signposting to and liaising with GPs and memory services, and tailoring action plans to embed new activities in existing routines and overcome future barriers which may arise due to memory difficulties.

## Conclusions

People with MCI have additional barriers to engaging with health promoting behaviour change interventions, beyond those experienced by older adults with normal cognition for their age. People may normalise their cognitive impairment as part of ageing, making them reluctant to address cognition within a health promotion intervention. Tailoring action plans to enable people to overcome barriers relating to memory and setting goals in line with existing health behaviours, routines, and priorities may increase engagement from older adults with MCI.

## Supplementary material

10.1136/bmjopen-2025-103314 online supplemental file 1

## Data Availability

Data are available on reasonable request.
