# Supplementary material for: Engagement with behaviour change in people with mild cognitive impairment and mild frailty: a qualitative study
Source: BMJ Open. 2026 May 4;16(5):e103314. doi: 10.1136/bmjopen-2025-103314 (PMC13141132; doi:10.1136/bmjopen-2025-103314)
Supplement: Supplementary file 2 [file bmjopen-2025-103314.reviewer_comments.pdf]

PEER REVIEW HISTORY

BMJ Open publishes all reviews undertaken for accepted manuscripts. Reviewers are asked to complete a checklist review form and are provided with free text boxes to elaborate on their assessment. These free text comments are reproduced below.

ARTICLE DETAILS

Title (Provisional)

Engagement with behaviour change in people with mild cognitive impairment and mild frailty: a qualitative study

Authors

Rookes, Tasmin Alanna; Frost, Rachael; Barrado-Martín, Yolanda; Catchpole, Jessica; Armstrong, Megan; Gardner, Benjamin; Gould, Rebecca L.; Cooper, Claudia; Hammond, Cecilia; Marston, Louise; Walters, Kate R

VERSION 1 - REVIEW

**Reviewer**	**1**
**Name**	**Donath, Carolin**
**Affiliation**	**Friedrich-Alexander University Erlangen-Nuremberg, Faculty of Medicine, Department of Psychiatry and Psychotherapy, Center for Health Services Research**
**Date**	**17-Apr-2025**
**COI**	**None**

Dear authors of the study „Barriers and facilitators to behaviour change in people with mild cognitive impairment: a qualitative study with older adults with mild frailty",

Thank you for submitting your interesting manuscript! Please find enclosed my suggestions to improve the article for the reader.

Title & Abstract

1. Having just read the title and the abstract only, I wonder if the matter concerns subjects with MCI or with frailty or both. I suggest to choose a title that is more unambiguous.
2. You mention the health promotion program that was designed to support older adults. Could you briefly include information through what components? What was the goal of the health promotion program? It could be everything – healthy diet, exercise, promotion of social activities…

3. The method part of the abstract is too short. It lecks information on when and where data were collected, also information on how or where the sample was recruited, the main method used for analysis and the basic description of the sample concerning MCI classification.
4. I understand well, what is meant with two themes that were extracted. However, the expression of "perceptions towards addressing…" is a somewhat complicated phrasing. Please revise for easier understanding.
5. I see as a further limitation that should be named at the end of the abstract, that only people from one health system (one country) were included. People living in other countries, even in the Western society, may face totally different barriers, depending if their health insurance system is for example dominantly privately (e.g. US) or a mixture of statutory health insurance and private health insurance (e.g. Germany).
6. I am not convinced that certain author team variables should be named as a strength of a study. Honestly, I expect from every study published in a quality journal with peer review that methodological rigor and careful interpretation is used. I suggest to delete this bullet point.

Introduction

7. Please present more recent epidemiological data than ones that are 10 years old.
8. Are the numbers in the UK comparable to other (at least) Western societies? Could you report that?
9. Second paragraph: the authors state that "there are no specific management recommendations for people with MCI". However, there are guidelines for people living with MCI from other Western countries, like a German S2e guideline or the American Practice Guideline for Mild Cognitive Impairment from the American Academy of Neurology (AAN). They published 2018 an Update and there are many recommendations on the management of MCI, also many non-pharmacological; thus, interventions that affect the lifestyle. I see no reason, why those recommendations should only make sense for American people living with MCI. So even if there is not a specific guideline of the NHS, clinicians and researchers should orient towards the available guidelines rather than not taking any measure or acting "intuitively". This knowledge base should be incorporated into the introduction.
10. Please provide a reference for the claimed result named in introduction lines 35, 36.
11. The only example study for health promotion programs is a study on fall prevention that is thematically not fitting. Please include studies that refer to health promotion factors published in the systematic review of Livingston et al. 2020 (your reference number 10) and are designed to reduce the risk for cognitive decline/dementia as you have argued in paragraphs 1 and 2 of the introduction.
12. On what theory was the intervention based? Transtheoretical model or Health Action Process Approach. There are many psychological models that are designed to predict behavior change and many of them are evaluated and have been the base of non-pharmacological interventions that promote a healthier lifestyle. Thus, there should not be an intervention nowadays that is developed without incorporation of the vastly available theories of health psychology.
13. What were the hypotheses concerning facilitators and barriers? Please state and report literature findings on studies that explored facilitators and barriers of implementing interventions for community-dwelling people of higher age.

Methods

14. I do not agree with the classification used for people with MCI. The author of the MoCA suggests the range of 22 to 26 raw points as interpretation of "Mild Cognitive Impairment,

further exploration needed”. Literature on revised cut-off approaches (Thomann et al.) suggest that people with more than 26 raw points “may be considered as not pathological with very high accuracy (91%)”. This implies, that your upper boundary for classification of potential MCI should rather be 26 than 25, since both the original authors as well as the validation of the cut-off – studies have underlined this cut-off-value. Even more critical is your lower boundary though. There is a high probability that people with a MoCA-score of 18 already suffer dementia and thus are classified wrong as having MCI. Thomann et al. suggest the cut-off of 23 or lower for classifying people that are very likely not healthy with a accuracy of 88%, including both mild and major neurocognitive disorder. Nasreddine et al. suggest the boundary of 21 points or lower for cognitive disfunction that is worse than MCI. Taking both references into account I cannot comprehend how the boundary of 18 points for potential MCI is arguable.

I strongly recommend that you reclassify your 29 participants, according to the literature. Especially those having 21 points or lower should be excluded from the analysis. It is a clinically relevant difference if a person has MCI or mild dementia.

15. Was the RCT registered? Please include the information somewhere in the method section.
16. Where did you recruit the sample? Only London? Where the people only living there in this metropolitan urban area? This might be a main interpretation fact concerning facilitation or barriers of health promotion. Many things are in urban areas easier accessible or available at all only there.
17. Did you test and potentially exclude people suffering from acute depression? A frequent symptom of depression is cognitive impairment. Different to MCI or dementia the cognitive impairments in depression are reversable. However, somebody with a depression suffering also from anhedonia, lack of motivation and depressed mood might judge a behavioral intervention differently than somebody without an affective disorder. This could have biased your results if you have not excluded those patients.
18. Did you have a structured or semi-structured interview? Could you name the asked questions (e.g. in the supplements/additional material) for both groups?
19. Did you calculate any indices for reliability of the ratings, like Cohen’s Kappa between the two raters TR and YBM? If not, why?

Results

20. For baseline demographics, always report arithmetic mean together with standard deviation. Please revise Table 1.
21. Legend of Table 1. Even if I know, how the Barthel-Index is scored, please include into the legend an interpretation aid (possible range: …; higher scores indicate …). The same for the socio-economic status. I have no idea, what is behind that score and if you build a sum score(?). Did somebody else publish this score already?
22. What is the explanation that the MoCA-score in your sample is higher 12 months later, even though it is known (and mentioned in your manuscript) that a certain number of individuals progress from MCI to dementia.
23. I would suggest to clearer summarize the facilitators and barriers for each of the three themes that were identified from the texts. For the reader it would help to have them written down at the end of the section of each theme.
24. I cannot see how really the content of the 29 interviews of the people living with MCI was included in the facts named under the results section. In how many of the 29 cases could you extract content of Theme 1, Theme 2 and Theme 3.?

25. The included quotes seem to be from a very small number of participants, some of them are quoted several times. It would help if each participant got additionally a number, like “participant 1, female, 73”. I would also prefer a broader range of quotes from different participants in the manuscript.

Discussion

26. I really like the practical relevance of the discussion! Could you also incorporate a section where you compare your results to the literature on barriers and facilitators (see my comment no. 13.)? Please, also refer to your hypotheses you had concerning the research question.
27. I would also suggest to make a link back to the theory of your intervention. In many models, like in the TTM, specific interventions are suggested for each stage of the behavioral change, also for the maintenance stage, that you mention for example in line 6 to 9 of page 12. How do the results you found are inline with the suggestions of the theoretical models?

Strength and Limitations

28. See my points no. 5 and 6

Conclusion

29. Needs to be reformulated. The second sentence is just a result and thus not belong here. Conclusions should be drawn from the essence of your results and potentially integrate some practical relevance and suggestion.

**Reviewer**	**2**
**Name**	**Nazar, Gabriela**
**Affiliation**	**Universidad de Concepción, Psychology**
**Date**	**12-Nov-2025**
**COI**	**None**

The study aims to explore barriers and facilitators to engagement with and the perceived benefit of behaviour change interventions for people with MCI, using the HomeHealth intervention as a case study from the perspective of those delivering and receiving the intervention.

In its rationale, it states that there are no specific management recommendations for people with MCI and that identifying and intervening with health promotion interventions when a individuals have MCI could prevent further decline to dementia.

The objective is unclear in how it's worded.

Please specify the characteristics from which participants were sampled. Only included the name of the variable, such as age, gender, ethnicity, geographic location, socioeconomic status, education level, or the type of goal(s) participants chose to set.

Please provide more details about who the "two public contributors with lived experience of MCI" were and what their roles were in the project.

Please provide a more detailed description of the thematic reflexive analysis technique.

The themes presented as results, although informative, do not align with the study's aim, namely to identify the barriers and facilitators to engagement with, and the perceived benefits of, behaviour change interventions for people with MCI. They should be stated in terms that allow the objective of the study to be answered.

VERSION 1 - AUTHOR RESPONSE

Reviewer	Comment	Response
Reviewer 1	Having just read the title and the abstract only, I wonder if the matter concerns subjects with MCI or with frailty or both. I suggest to choose a title that is more unambiguous.	Thank you for highlighting this point. The title has been updated to avoid ambiguity.
Reviewer 1	You mention the health promotion program that was designed to support older adults. Could you briefly include information through what components? What was the goal of the health promotion program? It could be everything – healthy diet, exercise, promotion of social activities…	Some additional information about the intervention has been added to the last paragraph of the introduction on page 4. The aim of the HomeHealth intervention can be seen at the end of the first paragraph in the abstract and in paragraph 4 of the introduction: “to support older adults with mild frailty to maintain their independence”.
Reviewer 1	The method part of the abstract is too short. It lacks information on when and where data were collected, also information on how or where the sample was recruited, the main method used for analysis and the basic description of the sample concerning MCI classification.	Unfortunately, the journal requirements only allow for 200 words in the abstract, so we have been limited by what we can include. We have tried to rephrase where we can , to add more methodological detail within this limit. The analysis is stated as ‘thematic analysis’ in paragraph 3 of the abstract.
Reviewer 1	I understand well, what is meant with two themes that were extracted. However, the expression of “perceptions towards addressing…” is a somewhat complicated phrasing.<br>Please revise for easier understanding.	This has been simplified to “Addressing memory as a goal”.
Reviewer 1	I see as a further limitation that should be named at the end of the abstract, that only people	We acknowledge differing health systems will provide different barriers; therefore, the 2nd bullet point of the

	from one health system (one country) were included. People living in other countries, even in the Western society, may face totally different barriers, depending if their health insurance system is for example dominantly privately (e.g. US) or a mixture of statutory health insurance and private health insurance (e.g. Germany).	strengths and limitations has been expanded to make these points explicitly clear.
Reviewer 1	I am not convinced that certain author team variables should be named as a strength of a study. Honestly, I expect from every study published in a quality journal with peer review that methodological rigor and careful interpretation is used. I suggest to delete this bullet point.	Thank you for this point. We have deleted this as suggested.
Reviewer 1	Please present more recent epidemiological data than ones that are 10 years old.	This has been updated to a 2019 reference, reference 6.
Reviewer 1	Are the numbers in the UK comparable to other (at least) Western societies? Could you report that?	We have added this in: *These estimates are in line with other European countries (8)* in page 3 paragraph 1.
Reviewer 1	Second paragraph: the authors state that “there are no specific management recommendations for people with MCI”. However, there are guidelines for people living with MCI from other Western countries, like a German S2e guideline or the American Practice Guideline for Mild Cognitive Impairment from the American Academy of Neurology (AAN). They published 2018 an Update and there are many recommendations on the management of MCI, also many non-pharmacological; thus, interventions that affect the	Thank you for highlighting this. The 2nd paragraph of the introduction, page 3 now opens with a reference to international guidelines. It has also been restructured to reinforce that HCPs are not implementing guidelines well enough and acknowledge that this is unlikely to bring about behaviour change.

	lifestyle. I see no reason, why those recommendations should only make sense for American people living with MCI. So even if there is not a specific guideline of the NHS, clinicians and researchers should orient towards the available guidelines rather than not taking any measure or acting "intuitively". This knowledge base should be incorporated into the introduction.	
Reviewer 1	Please provide a reference for the claimed result named in introduction lines 35, 36.	Thank you for highlighting this – we have added a reference.
Reviewer 1	The only example study for health promotion programs is a study on fall prevention that is thematically not fitting. Please include studies that refer to health promotion factors published in the systematic review of Livingston et al. 2020 (your reference number 10) and are designed to reduce the risk for cognitive decline/dementia as you have argued in paragraphs 1 and 2 of the introduction.	Thank you for this observation. We agree that the argument we were trying to make did not flow well. An additional sentence has been added to paragraph 3, page 3: *Many health promotion interventions have been delivered to people with MCI to improve their cognitive and physical functioning outcomes (17).*
Reviewer 1	On what theory was the intervention based? Transtheoretical model or Health Action Process Approach. There are many psychological models that are designed to predict behavior change and many of them are evaluated and have been the base of nonpharmacological interventions that promote a healthier lifestyle. Thus, there should not be an intervention nowadays that is developed without incorporation of the	The intervention was based on the COM-B behaviour change model, and this has now been added to the intervention description and referenced on page 4 paragraph 5.

	vastly available theories of health psychology.	
Reviewer 1	What were the hypotheses concerning facilitators and barriers? Please state and report<br>literature findings on studies that explored facilitators and barriers of implementing interventions for community-dwelling people of higher age.	As the approach to analysis was inductive, we did not have any prior hypotheses about the barriers and facilitators. As each intervention is unique, we were driven by the data and what participants reported. Our aim was to allow themes to emerge from participants' accounts rather than impose predefined assumptions. To address your request, we have ensured the findings have been compared to the wider older adult and MCI literature in the discussion, highlighting where our results align with or differ from previous studies in paras 2, 4, 5 on page 12, as per qualitative research methods best practice.
Reviewer 1	I do not agree with the classification used for people with MCI. The author of the MoCA<br>suggests the range of 22 to 26 raw points as interpretation of "Mild Cognitive Impairment, further exploration needed". Literature on revised cut-off approaches (Thomann et al.) suggest that people with more than 26 raw points "may be considered as not pathological with very high accuracy (91%)". This implies, that your upper boundary for classification of potential MCI should rather be 26 than 25, since both the original authors as well as the validation of the cut-off – studies have underlined this cut-off-value. Even more critical is your lower boundary though. There is a high probability that people with a MoCA-score of 18 already suffer dementia and thus are classified wrong as	We appreciate your detailed feedback and agree that the use of MoCA as a pragmatic screening tool for MCI is a limitation. Our decision was based on the need to identify individuals without a formal diagnosis and include diverse perspectives often excluded from research.<br>We acknowledge the variability in recommended cut-offs, with some literature suggesting 24 and others 26 for optimal sensitivity and specificity (Carson N, Leach L, Murphy KJ. A re-examination of Montreal Cognitive Assessment (MoCA) cutoff scores. International journal of geriatric psychiatry. 2018 Feb;33(2):379-88). An upper limit of 25 was therefore selected as a pragmatic compromise between these two upper cut points noted in the literature, that was likely to identify people with at least some mild cognitive impairment (i.e. increase our specificity for MCI vs normal cognition, compared to an upper limit of 26).

	having MCI. Thomann et al. suggest the cut-off of 23 or lower for classifying people that are very likely not healthy with a accuracy of 88%, including both mild and major neurocognitive disorder. Nasreddine et al. suggest the boundary of 21 points or lower for cognitive disfunction that is worse than MCI. Taking both references into account I cannot comprehend how the boundary of 18 points for potential MCI is arguable.<br>I strongly recommend that you reclassify your 29 participants, according to the literature. Especially those having 21 points or lower should be excluded from the analysis. It is a clinically relevant difference if a person has MCI or mild dementia.	Raising the lower limit substantially could risk excluding participants whose scores may reflect sociodemographic factors (e.g., education, language) rather than dementia, especially given this was a single time-point measure and not diagnostic.<br>To address your concern, we have expanded the limitations section to clarify this rationale and note the potential misclassification risk. While this approach may reduce clinical precision, we consider it a strength in capturing voices of underserved groups, which aligns with the study's aims, including those with MCI that may have greater impairments, close to those with mild dementia. Therefore, we chose to be as pragmatic and inclusive as possible, to ensure a wide range of views were considered, which are often excluded in research.<br><br>To address this point, we have expanded on this topic in the limitations section of the discussion page 13 para 3 to make the limitation clearer and to align with some of the points you have made.
Reviewer 1	Was the RCT registered? Please include the information somewhere in the method section.	This is in the introduction where the HomeHealth intervention is introduced (HomeHealth - ISRCTN54268283)
Reviewer 1	Where did you recruit the sample? Only London? Where the people only living there in this metropolitan urban area? This might be a main interpretation fact concerning facilitation or barriers of health promotion. Many things are in urban areas easier accessible or available at all only there.	Thank you for highlighting this oversight. The 3 regions participants were recruited from have been added to the methods eligibility section page 4.<br>*"Participants were recruited into the trial from three regions of England, London, Hertfordshire, and West Yorkshire."*

Reviewer 1	Did you test and potentially exclude people suffering from acute depression? A frequent symptom of depression is cognitive impairment. Different to MCI or dementia the cognitive impairments in depression are reversable. However, somebody with a depression suffering also from anhedonia, lack of motivation and depressed mood might judge a behavioral intervention differently than somebody without an affective disorder. This could have biased your results if you have not excluded those patients.	Thank you for raising this important point. Depression was not an exclusion criteria as part of this study. The trial was supporting people with mild frailty to engage with a health promotion intervention and this study was exploring the role of MCI.<br>In the interviews participants were able to discuss the role of their mental health in relation to their engagement with the intervention. As this is not a quantitative study looking at causality, we did not exclude people with depression from the interviews as we wanted to capture their experiences of engagement and cognitive impairment as well, and how this might influence engagement in people with MCI. The limitation of how other factors may have impacted MoCA scores such as sleep and mood is included in the strengths and limitations section, in para 3 page 13.<br>When looking at implementation of health promotion interventions for older people with mild frailty and co-existing MCI these would generally be holistic and not exclude those with depression, so we felt it was important to include this group and understand how the interventions work in this context as well as in those without depression.
Reviewer 1	Did you have a structured or semi-structured interview? Could you name the asked questions (e.g. in the supplements/additional material) for both groups?	Semi-structured interviews are stated in the data collection sub-headings of the methods. The types of questions asked are stated in the second paragraph of the data collection sub-heading, bottom of page 4, and top of page 5.
Reviewer 1	Did you calculate any indices for reliability of the ratings, like Cohen's Kappa between the two raters TR and YBM? If not, why?	We chose not to calculate reliability of ratings for the purpose of this paper. Conducting Cohen's Kappa scores or similar are not common practice for inductive qualitative thematic analysis, including codebook analysis. This approach prioritises depth and

		flexibility over quantifying our agreement, which would not align with the epistemological underpinnings of the method.
Reviewer 1	For baseline demographics, always report arithmetic mean together with standard deviation.<br>Please revise Table 1.	Thank you for pointing this out. A column has been added to include this in Table 1.
Reviewer 1	Legend of Table 1. Even if I know, how the Barthel-Index is scored, please include into the legend an interpretation aid (possible range: ...; higher scores indicate ...). The same for the<br>socio-economic status. I have no idea, what is behind that score and if you build a sum score(?). Did somebody else publish this score already?	We have deleted the Barthel row, as this was not a screening criteria for this analysis and was included in error. We have added a reference for IMD that outlines how it is calculated and included more detail in Table 1.<br><br>IMD is a UK government measure of deprivation that ranks geographical areas by relative deprivation using factors such as income, employment, health, education, housing, and crime (Department for Levelling Up, Housing & Communities. (2019). English indices of deprivation 2019. GOV.UK. https://www.gov.uk/government/statistics/english-indices-of-deprivation-2019).
Reviewer 1	What is the explanation that the MoCA-score in your sample is higher 12 months later, even though it is known (and mentioned in your manuscript) that a certain number of individuals progress from MCI to dementia.	With such a small sample this could be due to chance, and we feel this would be more appropriately examined in more detail quantitatively, so we have not reflected on this within the current paper. However, from the main trial analysis we know that cognitive score did not significantly improve in the intervention arm in comparison to the control arm (Walters K, Frost R, Avgerinou C, Kalwarowsky S, Goodman C, Clegg A, et al. Clinical and cost-effectiveness of a home-based health promotion intervention for older people with mild frailty in England: a multicentre, parallel-group, randomised controlled trial. Lancet Healthy Longev [Internet]. 2025 Feb 1;6(2). Available from: https://doi.org/10.1016/j.lanhl.2024.1

		00670), so this is unlikely to be due to the intervention itself improving cognition.
Reviewer 1	I would suggest to clearer summarize the facilitators and barriers for each of the three themes that were identified from the texts. For the reader it would help to have them written down at the end of the section of each theme.	Thank you for your suggestion. We considered this but feel rewriting the themes in this suggested way would be deductive rather than inductive. Instead of outlining this under each theme in the findings, the themes are brought together in the way suggested at the start of the discussion on page 11 and compared to the wider literature, which we hope provides clarity on this point.
Reviewer 1	I cannot see how really the content of the 29 interviews of the people living with MCI was included in the facts named under the results section. In how many of the 29 cases could you<br>extract content of Theme 1, Theme 2 and Theme 3.?	We have gone through and added in IDs for each participant to the included quotes under each theme and sub-theme, so that the spread of evidence can be seen. It is not in line with the qualitative thematic analysis to state the number of interviewees each theme relates to, but hopefully the addition of IDs helps to reflect the diversity of perspectives represented.
Reviewer 1	The included quotes seem to be from a very small number of participants, some of them are quoted several times. It would help if each participant got additionally a number, like "participant 1, female, 73". I would also prefer a broader range of quotes from different participants in the manuscript.	Thank you for highlighting this. As above, we have now added the IDs in. We have added some additional quotes to support some points and increase representation from more participants whilst remaining within the journal's word limit.
Reviewer 1	I really like the practical relevance of the discussion! Could you also incorporate a section where you compare your results to the literature on barriers and facilitators (see my comment no. 13.)? Please, also refer to your hypotheses you had concerning the research question.	Thank you for your comment – we are pleased you enjoyed the practical relevance of the discussion. As per qualitative methods, we did not have hypotheses. We have however referred to the wider literature on barriers and facilitators throughout the discussion on pages 11 and 12, rather than having a specific subsection on this.
Reviewer 1	I would also suggest to make a link back to the theory of your intervention. In many models,	We agree this was needed and have added this into the discussion in para 1, page 12:

	like in the TTM, specific interventions are suggested for each stage of the behavioral change, also for the maintenance stage, that you mention for example in line 6 to 9 of page 12. How do the results you found are inline with the suggestions of the theoretical models?	*This aligns with the COM-B model of behaviour change, which this intervention was based on, where psychological capability and social opportunity are necessary to enable people change their behaviour.*
Reviewer 1	Strength and Limitations<br>See my points no. 5 and 6	Please see responses to these comments.
Reviewer 1	Needs to be reformulated. The second sentence is just a result and thus not belong here. Conclusions should be drawn from the essence of your results and potentially integrate some practical relevance and suggestion.	Thank you for your feedback. We agree that the original sentence was more descriptive of results than appropriate for the conclusion. This sentence in the conclusion on page 14 has been rephrased, as suggested.
Reviewer 2	The objective is unclear in how it's worded.	Thank you for highlighting this point. The aim has been reworded to be clearer on page 4.
Reviewer 2	Please specify the characteristics from which participants were sampled. Only included the name of the variable, such as age, gender, ethnicity, geographic location, socioeconomic status, education level, or the type of goal(s) participants chose to set.	We have added some additional detail about the variables/characteristics in the eligibility sub-section of the methods section on page 4. We have made it clearer that, rather than purposively sampling for specific sub-groups, we wanted to get a 'diverse range' and differing scores across the sample of people randomised to the treatment arm for the intervention.
Reviewer 2	Please provide more details about who the "two public contributors with lived experience of MCI" were and what their roles were in the project.	We have amended this to make it clearer the public contributors had MCI. As per a request from the PPI members, we have not included any personal details or demographics. However, their input and roles are outlined in the data collection section on page 5.
Reviewer 2	Please provide a more detailed description of the thematic reflexive analysis technique.	The reflexive thematic analysis section has been rewritten in relation to more common language used for the method.
Reviewer 2	The themes presented as results, although informative, do	Thank you for pointing out this discrepancy. We decided we did not

	not align with the study's aim, namely to identify the barriers and facilitators to engagement with, and the perceived benefits of, behaviour change interventions for people with MCI. They should be stated in terms that allow the objective of the study to be answered.	want the results to lose their nuance, by being restricted to the labels of barriers and facilitators and recognise the results go beyond this. Therefore, we have slightly reworded the aim and title to ensure that it captures the breadth of the work.

VERSION 2 - REVIEW

Reviewer 2
Name Nazar, Gabriela
Affiliation Universidad de Concepción, Psychology
Date 22-Jan-2026
COI

While the themes provide valuable contextual insight into participants' experiences of living with mild cognitive impairment (MCI), they remain largely descriptive and are insufficiently connected to the study's stated focus on engagement with behaviour change.

In particular, Theme 1 presents meaningful accounts of cognitive and emotional challenges; however, the analytic link to behaviour change engagement (e.g., motivation, capacity, adherence, or decision-making) remains mostly implicit rather than explicitly interpreted.

More broadly, several of the themes and subthemes appear to function as topical headings rather than as analytic themes. For example, the subtheme "Impact of cognitive impairment on daily living" identifies a topic area but does not inform a central organising concept or that explains how or why these experiences matter in relation to behaviour change engagement. As outlined in Braun and Clarke's reflexive thematic analysis, themes should move beyond naming content to provide an interpretive account of patterned meaning within the data.

Similarly, Theme 3 ("Adapting Behaviour Change Interventions for MCI") would benefit from greater specificity. The theme title should clearly specify what adaptations were made, how participants experienced them, or why they were perceived as supportive (or not) for engagement.

VERSION 2 - AUTHOR RESPONSE

Reviewer 2 comment:

While the themes provide valuable contextual insight into participants' experiences of living with mild cognitive impairment (MCI), they remain largely descriptive and are insufficiently connected to the study's stated focus on engagement with behaviour change.

In particular, Theme 1 presents meaningful accounts of cognitive and emotional challenges; however, the analytic link to behaviour change engagement (e.g., motivation, capacity, adherence, or decision-making) remains mostly implicit rather than explicitly interpreted.

More broadly, several of the themes and subthemes appear to function as topical headings rather than as analytic themes. For example, the subtheme "Impact of cognitive impairment on daily living" identifies a topic area but does not inform a central organising concept or that explains how or why these experiences matter in relation to behaviour change engagement. As outlined in Braun and Clarke's reflexive thematic analysis, themes should move beyond naming content to provide an interpretive account of patterned meaning within the data.

Similarly, Theme 3 ("Adapting Behaviour Change Interventions for MCI") would benefit from greater specificity. The theme title should clearly specify what adaptations were made, how participants experienced them, or why they were perceived as supportive (or not) for engagement.

Response:

Thank you for the opportunity to revise the manuscript. We agree with the reviewers' comments that some of the themes are a little descriptive. We have updated some of the theme names and content to try to overcome this. Particularly:

- We have renamed the first sub-theme to refer to daily activities rather than daily living and linked within the engaging in social interactions. However, ultimately this theme does provide context for the later points.
- We have expanded the second sub-theme, on page 7, to have more evidence and quotes.
- We have renamed theme 2 to be in the context of the intervention and have renamed the two sub-themes to be in the context of motivation and requirements, to account for the patterned meaning of the data. The second sub-theme in theme 3 has also been rephrased, to provide more meaning and context, in line with this.

- We agree that the techniques sub-theme needed to more explicit and we have completely reworked this, with more explicit reference to behaviour change theory and techniques. As we refer to a lot of techniques, we do not feel adding them to the sub-theme name would be helpful, but we hope explicitly stating them in the text helps to overcome this challenge.

VERSION 3 - REVIEW

**Reviewer**	**2**
**Name**	**Nazar, Gabriela**
**Affiliation**	**Universidad de Concepción, Psychology**
**Date**	**27-Feb-2026**
**COI**	

Thank you very much for your response and for the revisions made to the thematic structure. The updated theme titles are clearer and more informative than in the previous version, particularly due to the inclusion and clarification of subthemes, which strengthens the analytical coherence of the findings.

Please revise the Abstract so that it accurately reflects the updated themes and their wording as presented in the Results section. For example, the wording of Theme 2 differs.

VERSION 3 - AUTHOR RESPONSE

Reviewer: 2
Dr. Gabriela Nazar, Universidad de Concepción
Comments to the Author:
Thank you very much for your response and for the revisions made to the thematic structure. The updated theme titles are clearer and more informative than in the previous version, particularly due to the inclusion and clarification of subthemes, which strengthens the analytical coherence of the findings.
Please revise the Abstract so that it accurately reflects the updated themes and their wording as presented in the Results section. For example, the wording of Theme 2 differs.

Response: Thank you for your attention to detail. The theme 2 name in the abstract has been updated to reflect the new naming.
